# Rubisco Activases: AAA+ Chaperones Adapted to Enzyme Repair

**DOI:** 10.3389/fmolb.2017.00020

**Published:** 2017-04-10

**Authors:** Javaid Y. Bhat, Gabriel Thieulin-Pardo, F. Ulrich Hartl, Manajit Hayer-Hartl

**Affiliations:** Department of Cellular Biochemistry, Max-Planck-Institute of BiochemistryMartinsried, Germany

**Keywords:** Rubisco, Rubisco activase, AAA+ protein, CO_2_ fixation, photosynthesis

## Abstract

Ribulose-1,5-bisphosphate carboxylase/oxygenase (Rubisco), the key enzyme of the Calvin-Benson-Bassham cycle of photosynthesis, requires conformational repair by Rubisco activase for efficient function. Rubisco mediates the fixation of atmospheric CO_2_ by catalyzing the carboxylation of the five-carbon sugar ribulose-1,5-bisphosphate (RuBP). It is a remarkably inefficient enzyme, and efforts to increase crop yields by bioengineering Rubisco remain unsuccessful. This is due in part to the complex cellular machinery required for Rubisco biogenesis and metabolic maintenance. To function, Rubisco must undergo an activation process that involves carboxylation of an active site lysine by a non-substrate CO_2_ molecule and binding of a Mg^2+^ ion. Premature binding of the substrate RuBP results in an inactive enzyme. Moreover, Rubisco can also be inhibited by a range of sugar phosphates, some of which are “misfire” products of its multistep catalytic reaction. The release of the inhibitory sugar molecule is mediated by the AAA+ protein Rubisco activase (Rca), which couples hydrolysis of ATP to the structural remodeling of Rubisco. Rca enzymes are found in the vast majority of photosynthetic organisms, from bacteria to higher plants. They share a canonical AAA+ domain architecture and form six-membered ring complexes but are diverse in sequence and mechanism, suggesting their convergent evolution. In this review, we discuss recent advances in understanding the structure and function of this important group of client-specific AAA+ proteins.

## Introduction

**Figure 1:**
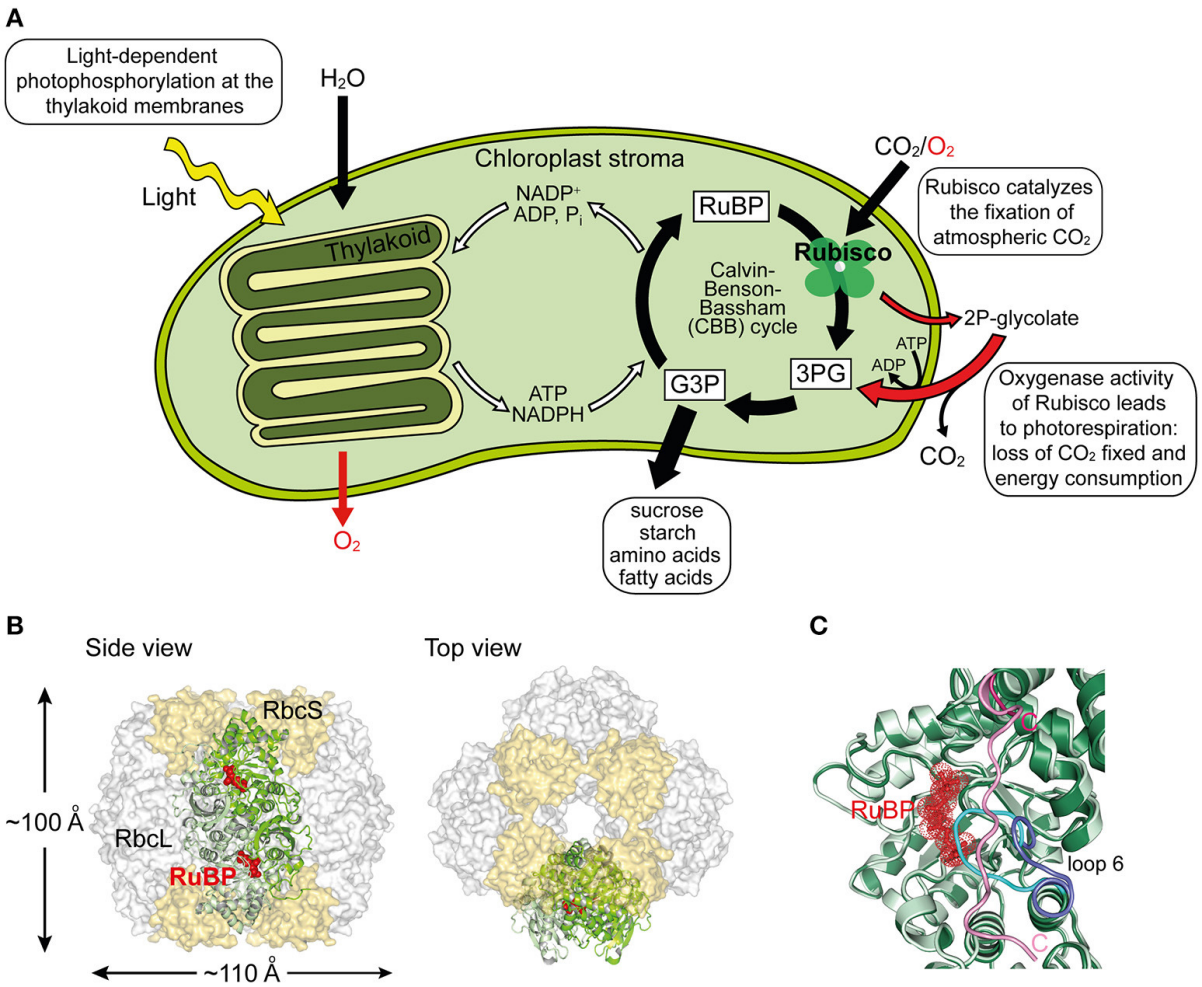
**Structure and function of Rubisco. (A)** Schematic depiction of photosynthesis in chloroplasts and the role of Rubisco. The light reaction and Calvin–Benson–Bassham (CBB) cycle of CO_2_ fixation, as well as the side-reaction of photorespiration are shown. RuBP, ribulose-1,5-bisphosphate; 3PG, 3-phosphoglycerate; G3P, glyceraldehyde-3-phosphate; 2P-glycolate, 2-phosphoglycolate. **(B)** Structure of hexadecameric form I Rubisco. Side and top views of Rubisco are shown in surface representation (PDB: 1RCX, Taylor and Andersson, 1997). One antiparallel RbcL dimer with RuBP bound in the active sites is shown in ribbon representation. **(C)** Superposition of open and closed conformations (PDB: 1RXO and 1RCX, respectively; Taylor and Andersson, 1997) of Rubisco. In the closed state (dark green), loop 6 (cyan) covers the active site, trapping the bound RuBP (red), and is pinned down by the flexible C-terminal peptide (pink) that stretches across the RbcL subunit. In the open conformation (pale green), loop 6 (dark blue) is retracted and the C-terminal peptide (pink) is disordered.

Ribulose-1,5-bisphosphate carboxylase/oxygenase (Rubisco) is the central enzyme of the Calvin-Benson-Bassham (CBB) cycle of photosynthesis (Figure 1A). Rubisco catalyzes the carboxylation of one molecule of ribulose-1,5-bisphospate (RuBP) and produces two molecules of 3-phosphoglycerate (3PG), which are then used for the synthesis of sugars, starch, amino acids, and fatty acids (Miziorko and Lorimer, 1983). As such, Rubisco is responsible for the overwhelming majority of carbon fixation by photoautotrophic organisms in the oceans and on land (Field et al., 1998). However, the specificity of Rubisco for CO_2_ is limited and the enzyme can also use oxygen as a substrate (Whitney et al., 2011). In this reaction, referred to as photorespiration, Rubisco catalyzes the oxygenation of RuBP, producing only one molecule of 3PG and one molecule of the toxic by-product 2-phosphoglycolate (2P-glycolate) (Figure 1A). 2P-glycolate must then be recycled into 3PG through an ATP-dependent mitochondrial-peroxisomal pathway with the loss of CO_2_. Photorespiration has long been regarded as a wasteful process, but recent advances suggest that it might play a crucial role in other aspects of plant life, including nitrate assimilation (Bloom, 2015; Hagemann and Bauwe, 2016; Walker et al., 2016). Moreover, Rubisco is a notoriously inefficient enzyme, with a very slow turnover, fixing at best only 10 CO_2_ molecules per second (Feller et al., 2008). As a consequence of its shortcomings, Rubisco amounts to ~50% of protein in plant leaves and is considered one of the most abundant proteins in nature (Ellis, 1979).

**Figure 2:**
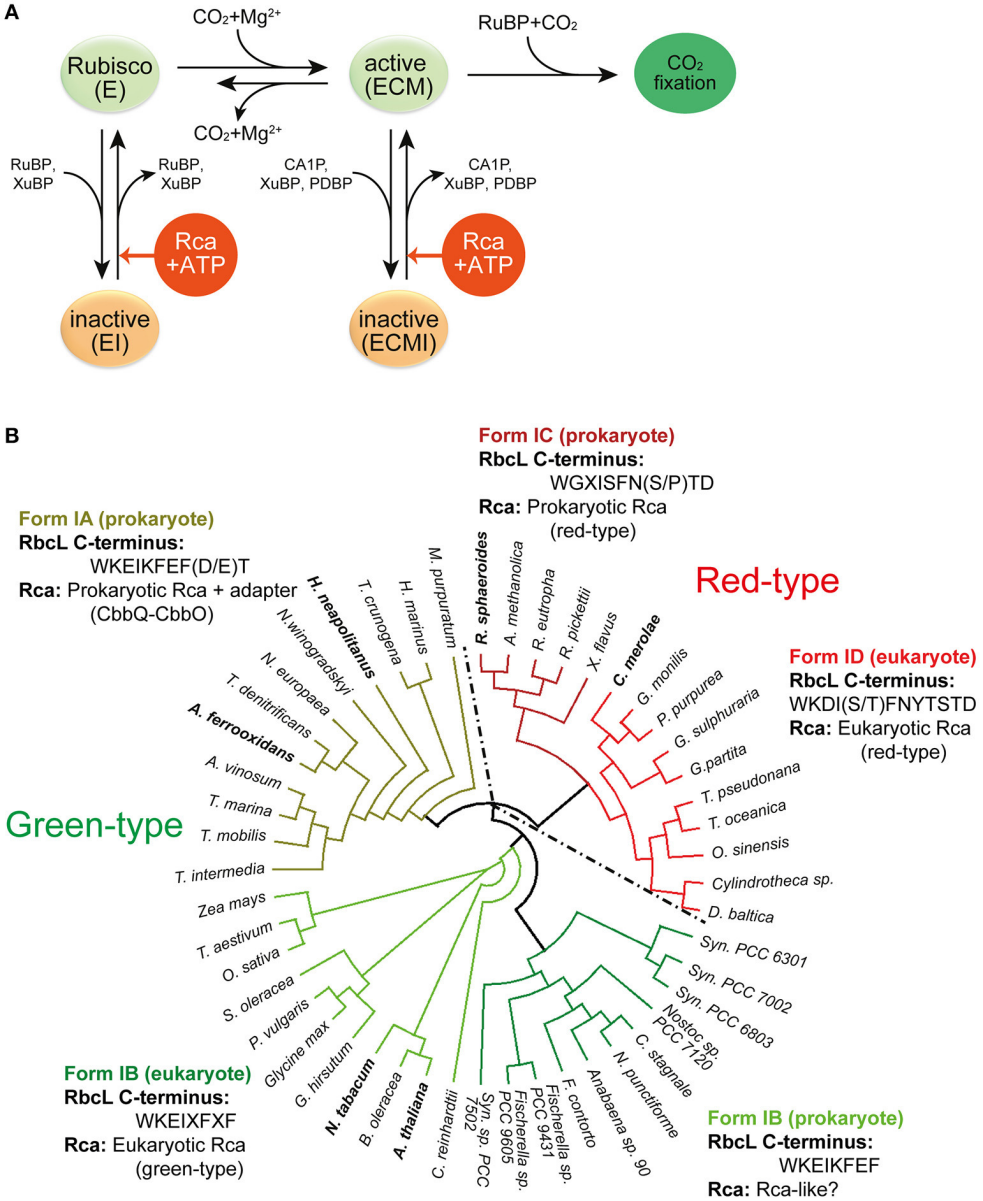
**Rubisco regulation by Rca. (A)** Regulation of Rubisco activity and inhibition by sugar phosphates. E, the non-carbamylated enzyme; ECM, the carbamylated and Mg^2+^ ion-bound enzyme; EI, the sugar phosphate inhibited E form; ECMI, the inhibited ECM form; Rca, Rubisco activase. Figure reproduced from reference Bracher et al. (2017). **(B)** Phylogenetic tree of selected Rubisco RbcL sequences. The green-type enzymes encompass form IA and IB, and the red-type enzymes form IC and ID. The RbcL C-terminal sequences and their associated Rca's are indicated. X represents variable residues. Rca's from species indicated in bold have been characterized biochemically and/or structurally and are described in this review. The phylogenetic tree was calculated by multiple sequence alignment using T-Coffee (Notredame et al., 2000) and the diagram was generated by the software Dendroscope (Huson and Scornavacca, 2012). Form IA (prokaryote): *M. purpuratum, Marichromatium purpuratum; H. marinus, Hydrogenovibrio marinus; T. crunogena, Thiomicrospira crunogena; H. neapolitanus, Halothiobacillus neapolitanus; N. winogradskyi, Nitrobacter winogradskyi; N. europaea, Nitrosomonas europaea; T. denitrificans, Thiobacillus denitrificans; A. ferrooxidans, Acidithiobacillus ferrooxidans; A. vinosum, Allochromatium vinosum; T. marina, Thiocapsa marina; T. mobilis, Thioflavicoccus mobilis; T. intermedia, Thiomonas intermedia*. Form IB (eukaryote): *Z. mays, Zea mays; T. aestivum, Triticum aestivum; O. sativa, Oryza sativa; S. oleracea, Spinacia oleracea; P. vulgaris, Phaseolus vulgaris; G. hirsutum, Gossypium hirsutum; N. tabacum, Nicotiana tabacum; B. oleracea, Brassica oleracea; A. thaliana, Arabidopsis thaliana*. Form IB (prokaryote): *C*. *reinhardtii, Chlamydomonas reinhardtii; Syn. PCC7502, Synechococcus sp. PCC 7502; F. contorta, Fortiea contorta; N. punctiforme, Nostoc punctiforme; C. stagnale, Cylindrospermum stagnale; Syn. PCC6803, Synechocystis PCC6803; Syn. PCC7002, Synechococcus PCC7002; Syn. PCC6301, Synechococcus PCC6301*. Form ID (eukaryote): *D. baltica, Durinskia baltica; O. sinensis, Odontella sinensis; T. oceanica, Thalassiosira oceanica; T. pseudonana, Thalassiosira pseudonana; G. partita, Galdieria partita; G. sulphuraria, Galdieria sulphuraria; P. purpurea, Porphyra purpurea; G. monilis, Griffithsia monilis; C. merolae, Cyanidioschyzon merolae*. Form IC (prokaryote): *X*. *flavus, Xanthobacter flavus; R. pickettii, Ralstonia pickettii; R. eutropha, Ralstonia eutropha; A. methanolica, Acidomonas methanolica; R. sphaeroides, Rhodobacter sphaeroides*.

The most common form of Rubisco, form I, found in plants, algae, cyanobacteria, and proteobacteria, is a ~550 kDa complex composed of eight large (RbcL, ~50–55 kDa) and eight small subunits (RbcS, ~15–20 kDa). The RbcL subunits are arranged as a toroid of antiparallel dimers that is capped at both ends by four RbcS subunits (Andersson and Backlund, 2008) (Figure 1B). To reach catalytic competence, one active site lysine of Rubisco (Lys201 using the *Nicotiana tabacum* nomenclature) must first be carboxylated by a non-substrate CO_2_ molecule, followed by the binding of a Mg^2+^ ion (Cleland et al., 1998). This process is called carbamylation and serves to position the substrate RuBP for efficient electrophilic attack by the second CO_2_ molecule that will be fixed in the CBB cycle (Andersson, 2008). Upon RuBP binding, the active site is closed via two sequential conformational changes in RbcL: Loop 6 in the C-terminal domain of RbcL extends over the bound RuBP trapping it below; the C-terminal tail of RbcL then stretches across the subunit and pins down loop 6, closing the active site (Bracher et al., 2017) (Figure 1C). Carbamylation of the apo form of the enzyme (“E”) to active Rubisco (“ECM”) is spontaneous (Figure 2A), but can only occur when the active site is in the open conformation.

Premature binding of RuBP to the apo form leads to the formation of a closed, inhibited enzyme (“EI”), in which the bound RuBP is unable to react with either CO_2_ or O_2_. Spontaneous decarbamylation followed by RuBP binding may occur during ongoing photosynthesis, also leading to loss of enzyme activity (“fallover”) (Zhu and Jensen, 1991). Moreover, Rubisco is inhibited by so-called misfire by-products, such as xylulose-1,5-bisphosphate (XuBP) and 2,3-pentodiulose-1,5-bisphosphate (PDBP), which are generated at a low frequency during the multistep catalytic reaction (Parry et al., 2008) (Figure 2A). Likewise, the inhibitor 2-carboxy-D-arabinitol-1-phosphate (CA1P), which is synthesized by some plants under low light conditions (also referred to as “night-time” inhibitor), inactivates the active form of Rubisco (Parry et al., 2008; Andralojc et al., 2012) (Figure 2A). In all these cases the closed, inhibited Rubisco (EI' or “ECMI”) reactivates only slowly, limited by the spontaneous rate of opening of the active site (Figure 1C).

Release of inhibitor from inactive Rubisco at a biologically relevant timescale is made possible through intervention by Rubisco activase (Rca) (Figure 2A). Rca enzymes belong to the AAA+ protein superfamily (Neuwald et al., 1999) and use ATP-driven conformational changes to remodel Rubisco, thereby facilitating the release of the inhibitory sugar phosphates (Portis, 2003; Portis et al., 2008). Since the discovery, in the early 1980's, of the first Rca in a photosynthesis mutant of *Arabidopsis thaliana* (Portis and Salvucci, 2002), Rca enzymes have been identified in many photosynthetic organisms containing either green-type or red-type Rubiscos, from chemoautotrophic bacteria to higher plants (Mueller-Cajar et al., 2011; Sutter et al., 2015; Tsai et al., 2015; Loganathan et al., 2016) (Figure 2B). Although displaying considerable sequence variability, all Rca's share the core subunit architecture of AAA+ proteins, consisting of a N-terminal nucleotide binding domain with α/β Rossman fold and a C-terminal α-helical domain (Hanson and Whiteheart, 2005; Snider et al., 2008; Wendler et al., 2012). Like most AAA+ proteins, the Rca enzymes function as hexameric donut-shaped rings, with their central pore implicated in threading specific peptides of Rubisco (Hauser et al., 2015; Bracher et al., 2017).

In this review, we will discuss recent advances in understanding the structure and mechanism of Rca's from the red and green lineages of photosynthetic organisms. The diversity of these enzymes provides a fascinating example of convergent evolution, and reflects the constraints under which Rca's and their cognate Rubisco substrates may have co-evolved.

## Rubisco activase of red-type rubisco form IC and ID

**Figure 3:**
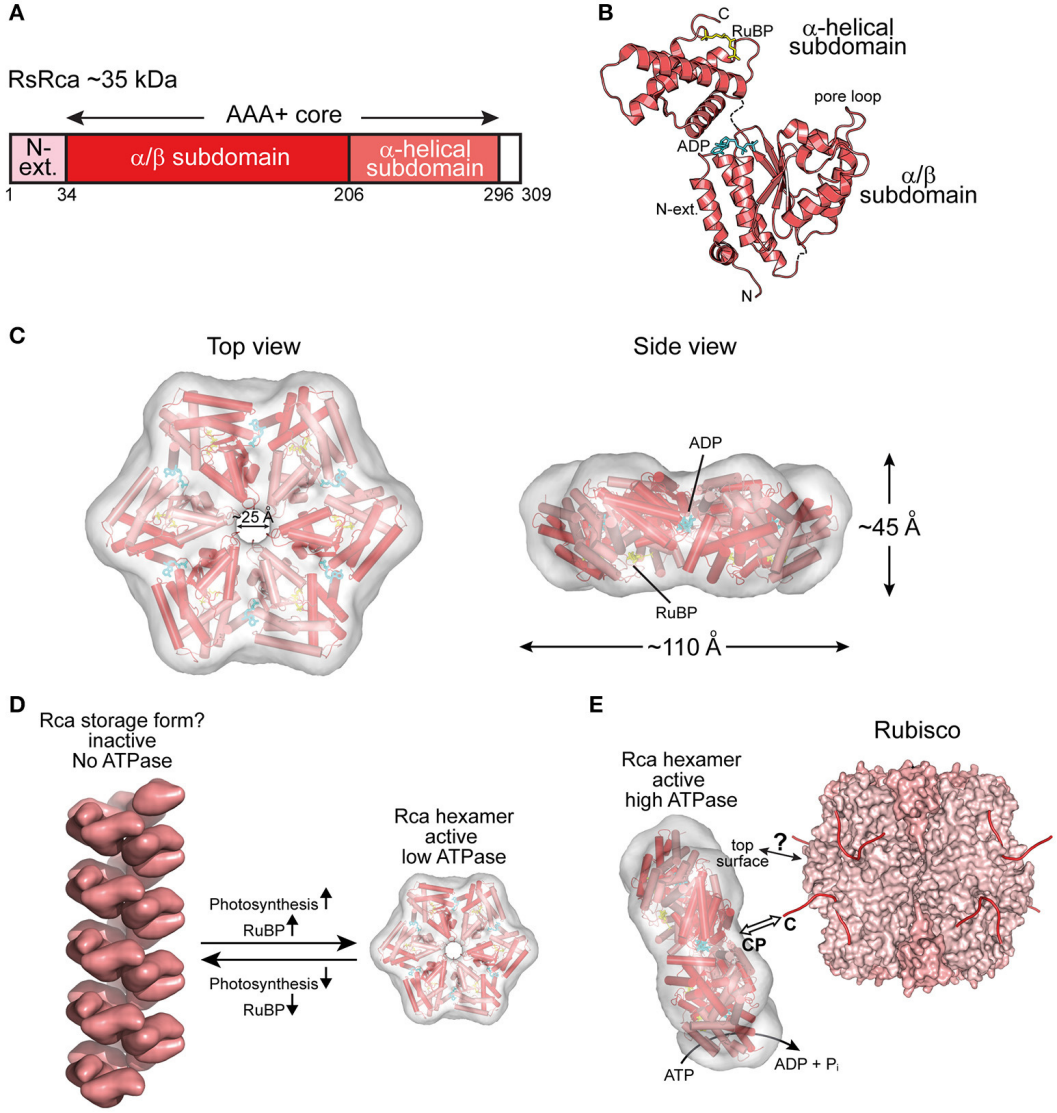
**The prokaryotic Rca of red-type form IC Rubisco. (A)** Schematic representation of the domain structure of Rca from *R. sphaeroides*. **(B)** Crystal structure of the monomer (PDB: 3SYL, Mueller-Cajar et al., 2011) shown in ribbon representation. The α/β and α-helical subdomains of the AAA+ core are indicated, as well as the N-terminal extension (N-ext.) of RsRca. The positions of the canonical pore loop, ADP (cyan) and the allosteric regulator, RuBP (yellow), are also indicated. **(C)** Top and side views of the RsRca hexameric model superposed on the electron microscopic reconstruction, with alternating subunits shown in two shades of red (EMDB EMD-1932; PDB 3ZUH, Mueller-Cajar et al., 2011). **(D)** Model of the putative storage form of prokaryotic Rca (Mueller-Cajar et al., 2011) from red-type form IC and its conversion to active hexamer. In the absence of photosynthetic activity (dark period), the concentration of free RuBP is low and Rca populates a helical assembly with no ATPase activity, avoiding unnecessary ATP consumption. Activation of photosynthesis results in the accumulation of free RuBP, reaching millimolar concentration (Von Caemmerer and Edmondson, 1986). Free RuBP binds to Rca, inducing its rearrangement to the catalytically competent hexamer. **(E)** Model of the mechanism of prokaryotic Rca from red-type form IC Rubisco. The active Rca hexamer interacts with inhibited Rubisco via its highly conserved top surface and concomitantly transiently pulls the extended C-terminal tail of the RbcL subunit into the central pore (CP). This action is mediated by the ATPase activity of Rca and results in the destabilization of the Rubisco active site, releasing the inhibitory sugar phosphate. Rca is displayed as in **(C)**. Rubisco (PDB: 4F0K, Stec, 2012) is shown in surface representation with the RbcL and RbcS subunits in different shades of pink. The RbcL C-termini are drawn as lines in red.

Rca has been known since the 1980s (Portis and Salvucci, 2002) but was assumed to be restricted to plants. The first prokaryotic Rca was only recently discovered in the proteobacterium *Rhodobacter sphaeroides*, which contains the red-type Rubisco form IC (Mueller-Cajar et al., 2011) (Figure 2B). RsRca is encoded by the *cbbX* gene located immediately downstream of the *rbcL* and *rbcS* genes (Gibson and Tabita, 1997). Inactivation of *cbbX* in *R. sphaeroides* resulted in impaired photoautotrophic growth at low CO_2_ levels. The structural and functional analysis of RsRca provided critical insights into the mechanism of Rubisco remodeling. The RsRca subunit (~35 kDa) is composed of the AAA+ core module with a compact α-helical extension at the N-terminus (Mueller-Cajar et al., 2011) (Figures 3A,B). The two subdomains of the core module are separated by a short flexible linker. The α*/*β subdomain harbors the characteristic Walker A and B nucleotide binding motifs (Mueller-Cajar et al., 2011; Bracher et al., 2017).

The active hexameric complex of RsRca forms only in the presence of ATP and RuBP, the substrate of its target enzyme Rubisco. The RuBP binding site is located in the α-helical subdomain at the bottom of the hexamer (Figures 3B,C). The hexamer exhibits a ~25 Å wide central channel lined by “canonical” pore loop residues (Tyr/Ile/Gly) (Mueller-Cajar et al., 2011). In the absence of RuBP, RsRca forms spiral-shaped high molecular weight assemblies that are largely ATPase inactive and may represent a storage form when the organism is not photosynthetically active (Mueller-Cajar et al., 2011) (Figure 3D). Thus, the generation of RuBP during photosynthesis would induce the conversion of this storage form into functional hexamers (Figure 3D). Biochemical and mutational analysis showed that remodeling of Rubisco depends on the canonical pore loops and the conserved top surface of the hexamer (Mueller-Cajar et al., 2011). Moreover, reactivation of *R. sphaeroides* Rubisco required the intact C-terminal sequence of RbcL, which is extended in red-type Rubiscos by ~5–10 residues relative to green-type RbcL. Binding to inhibited Rubisco stimulates the ATPase activity of RsRca ~4-fold (Mueller-Cajar et al., 2011), in a manner dependent on both the RbcL C-terminus and the top surface of the RsRca hexamer. These findings suggest that RsRca docks onto Rubisco with its top surface and the pore loops transiently pull the C-terminal tail of RbcL into the central pore, to facilitate opening of the active site pocket and release the inhibitory sugar phosphate (Figure 3E). This mechanism resembles the threading of ssrA-tagged proteins through the central pore of the bacterial ClpX for degradation by the ClpP protease (Olivares et al., 2016).

Interestingly, the red alga *Cyanidioschyzon merolae*, containing Rubisco form ID (Figure 2B), has two *cbbX* genes, one nuclear-encoded and one plastid-encoded (Loganathan et al., 2016). It was recently shown that the functional CmRca is a 1:1 hetero-hexamer between nuclear- and plastid-encoded subunits (Loganathan et al., 2016). Both of these Rca subunits share 60–70% identity with RsRca. In the case of CmRca, RuBP acts as an allosteric regulator for modulation of the ATPase activity but is not required for hexamer formation (Loganathan et al., 2016). In both the red-type prokaryotic and eukaryotic Rca enzymes, RuBP regulation of the ATPase activity provides a link between the functional state of the CBB cycle and Rubisco activity.

## Prokaryotic rubisco activase of green-type rubisco form IA

**Figure 4:**
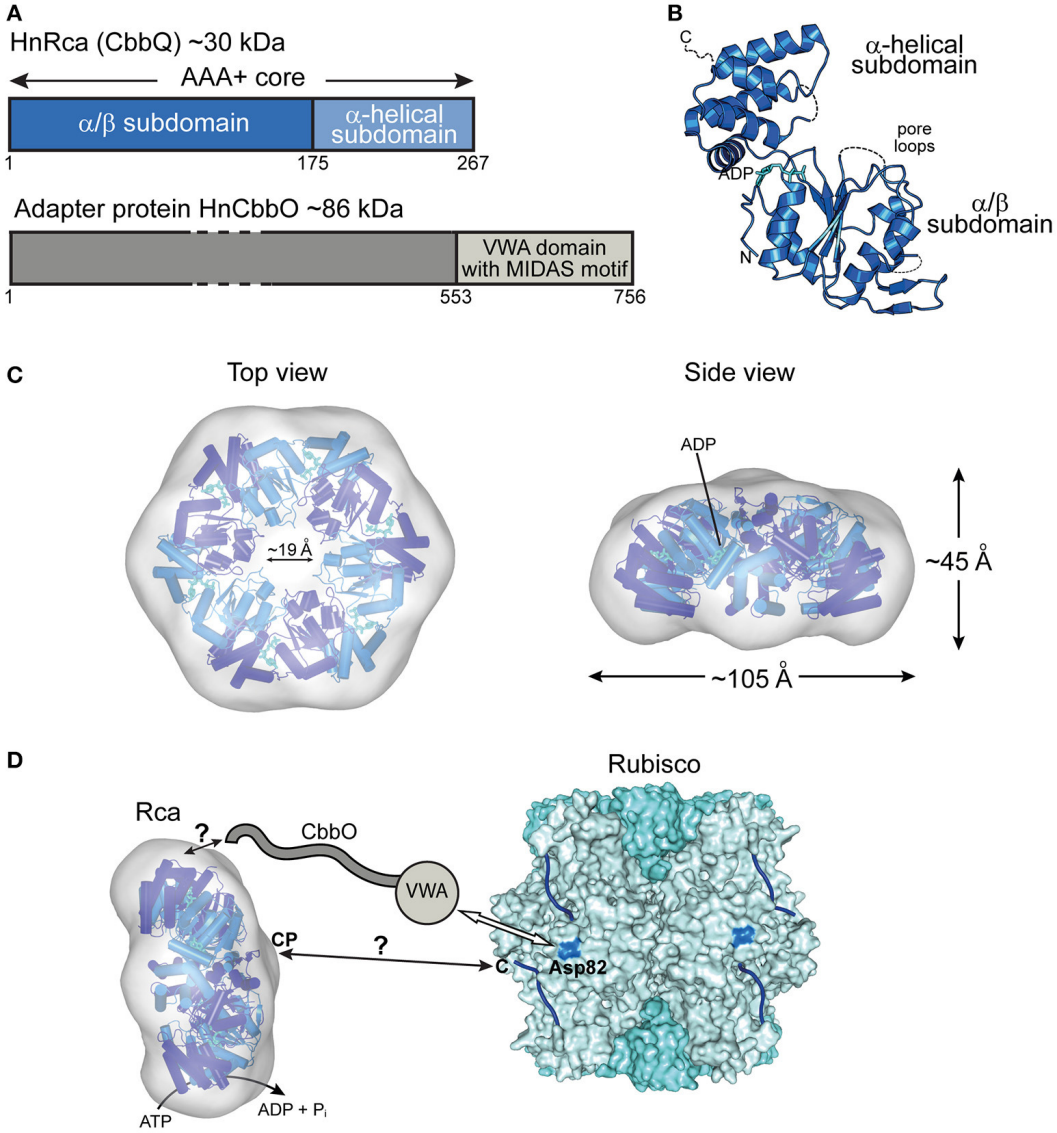
**The prokaryotic Rca of the green-type form IA Rubisco. (A)** Schematic representation of the domain structure of Rca from *H. neapolitanus* and its adapter protein (CbbO). **(B)** Crystal structure of the monomer (PDB: 5C3C, Sutter et al., 2015) shown in ribbon representation. The α/β and α-helical subdomains of the AAA+ core are indicated, as well as the positions of the pore loops and ADP (cyan). **(C)** Top and side views of the HnRca hexameric model (PDB: 5C3C, Sutter et al., 2015) superposed on the electron microscopic reconstruction of the Rca hexamer from *A. ferrooxidans* (EMDB: EMD-6477, Tsai et al., 2015). Alternating subunits shown in two shades of blue. **(D)** Model of the mechanism of prokaryotic Rca from green-type form IA Rubisco. The Rca hexamer interacts with inhibited Rubisco via the VWA domain of its adapter protein CbbO, recognizing the exposed acidic residue Asp82 (marine blue) on the RbcL subunit of Rubisco. Whether the central pore (CP) then engages the C-terminal tail of the RbcL subunit, remains unclear. The hexameric HnRca is displayed as in **(C)**. Rubisco (PDB: 1SVD, Kerfeld et al., 2004) is shown in surface representation with the RbcL and RbcS subunits in different shades of blue. The RbcL C-termini are represented by blue lines.

The most recent addition to the family of activases are the *cbbQ/cbbO* genes from the chemoautotrophic bacteria *Acidithiobacillus ferrooxidans* and *Halothiobacillus neapolitanus*, containing the green-type Rubisco form IA (Sutter et al., 2015; Tsai et al., 2015) (Figure 2B). These genes are generally associated with the Rubisco operon, with the *cbbQ* gene encoding the ~30 kDa AAA+ subunits and the *cbbO* gene a Rubisco adaptor protein of ~82–88 kDa. Structural and biochemical characterization showed that these proteins function as bipartite complexes consisting of the hexameric CbbQ activase (AfRcaI; HnRca) with CbbO as a co-factor (Sutter et al., 2015; Tsai et al., 2015) (Figure 4A). The α/β subdomain of AfRcaI and HnRca belong to the MoxR group of prokaryotic AAA+ proteins (Figures 4B,C), which often cooperate with proteins that contain the von Willebrand factor A (VWA) domain (Wong and Houry, 2012). Indeed, CbbO has a VWA domain with a typical metal-ion-dependent adhesion site (MIDAS), a motif usually involved in protein-protein interactions via a cation (generally Mg^2+^) (Whittaker and Hynes, 2002) (Figure 4A). Mutagenesis showed that the MIDAS motif interacts with aspartate 82 of the RbcL subunit of *A. ferrooxidans* (Tsai et al., 2015) (Figure 4D). Similar to the synergistic ATPase activation of RsRca and CmRca by RuBP and the inhibited Rubisco (Mueller-Cajar et al., 2011; Loganathan et al., 2016), the ATPase activity of AfRcaI is stimulated by the binding of both CbbO and the inhibited Rubisco (Tsai et al., 2015). This suggests that a two-step conformational change in the activase hexamer leads to optimal ATPase activity for Rubisco reactivation.

Furthermore, deletion or alanine substitution of the last two residues of the C-terminal tail of form IA RbcL resulted in loss of AfRcaI/CbbOI-mediated reactivation of inhibited Rubisco (Tsai et al., 2015). This suggests that the interaction of AfRcaI with the RbcL C-terminus is functionally critical, similar to the mechanism of red-type Rca described above. However, AfRcaI and HnRca do not have the canonical pore loop residues known to be involved in threading of flexible sequences into the central pore (Hanson and Whiteheart, 2005; Olivares et al., 2016). Accordingly, mutating these residues did not result in loss of function (Tsai et al., 2015). In the current model, CbbO acts as an adapter between the activase and Rubisco. Whether and how a pulling force is involved in remodeling remains to be investigated.

Interestingly, *A. ferrooxidans* also contains a form II Rubisco operon associated with a second pair of *cbbQ2/cbbO2* genes (Tsai et al., 2015). The well-characterized form II Rubisco of the α-proteobacterium *Rhodospirullum rubrum* is a dimer of only RbcL subunits and is Rca-independent (Jordan and Chollet, 1983; Pearce, 2006). The form II Rubisco of *A. ferrooxidans* is a trimer of RbcL_2_ units that can undergo inhibition by tightly binding sugar phosphates (Tsai et al., 2015). Reactivation requires the interaction with AfRcaII/CbbOII (Tsai et al., 2015), providing the first evidence for a Rca-dependent form II Rubisco.

## Eukaryotic rubisco activase of green-type rubisco form IB

**Figure 5:**
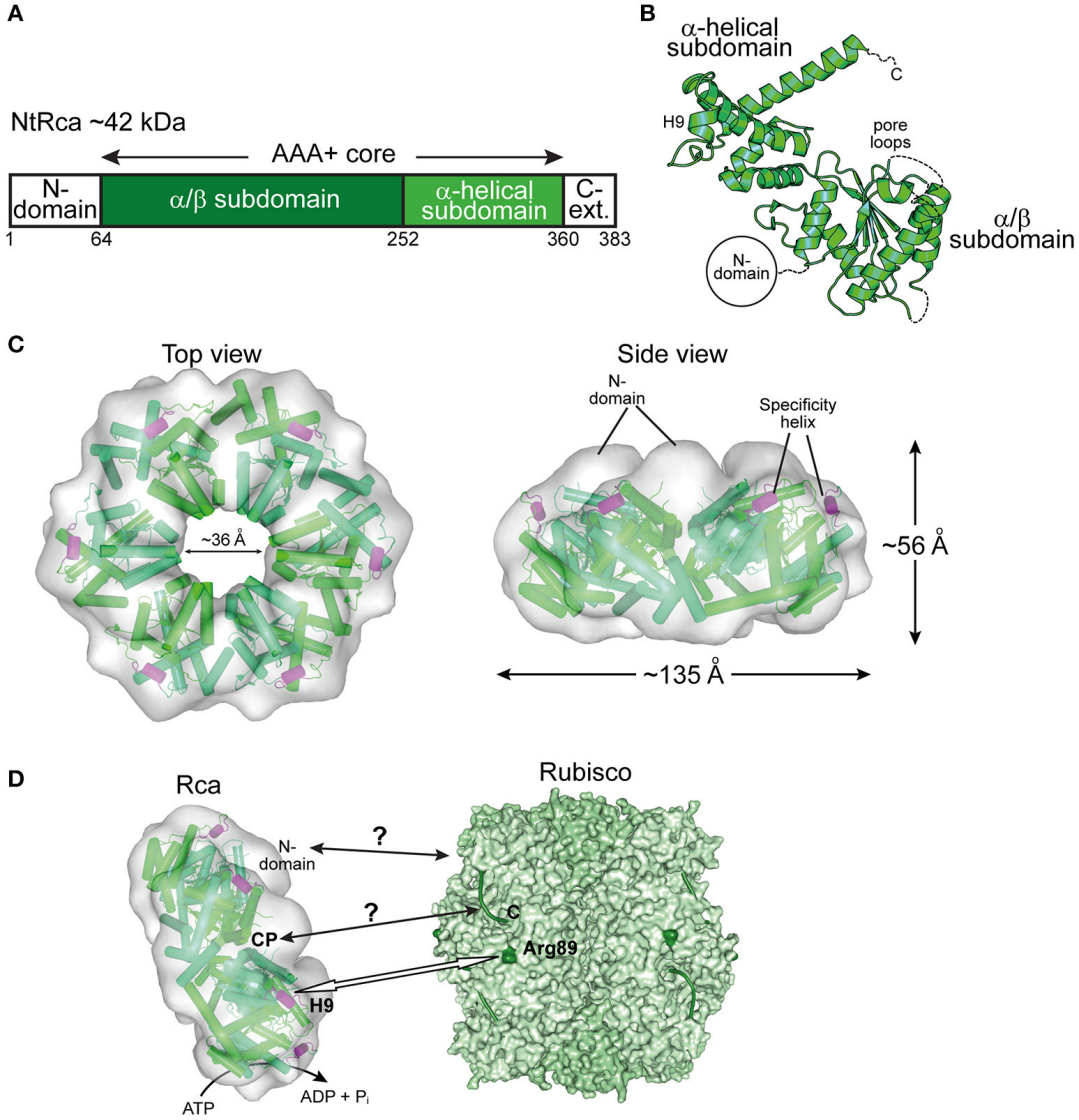
**The eukaryotic Rca of the green-type form IB Rubisco. (A)** Schematic representation of the domain structure of Rca from *N. tabacum*. **(B)** Crystal structure of the monomer (PDB: 3T15, Stotz et al., 2011) shown in ribbon representation. The α/β and α-helical subdomains of the AAA+ core are indicated, as well as the positions of the pore loops and the specificity helix H9. The positions of the N-terminal domain (N-domain) and the flexible C-terminal extension (C-Ext.), not present in the crystallized construct, are also indicated. **(C)** Top and side views of the NtRca hexameric model (PDB: 3ZW6, Stotz et al., 2011) superposed on the electron microscopic reconstruction (EMDB: EMD-1940, Stotz et al., 2011). The unfilled electron density at the top of the hexamer probably represents the N-domains. Alternating subunits are shown in two shades of green and the specificity helix (H9) in purple. **(D)** Model of the mechanism of eukaryotic Rca from green-type form IB Rubisco. The Rca hexamer interacts with inhibited Rubisco via the N-domain and H9 recognizes the exposed basic residue Arg89 (dark green) on the RbcL subunit. Whether the central pore (CP) engages the C-terminal tail of RbcL, remains unclear. The hexameric NtRca is displayed as in **(C)**. Rubisco (PDB: 1EJ7, Duff et al., 2000) is shown in surface representation with the RbcL and RbcS subunits in different shades of green. The RbcL C-termini are shown as green lines.

Almost three decades after the discovery of Rca in *A. thaliana* (Portis and Salvucci, 2002; Portis, 2003), the first crystal structures of Rca for eukaryotic green-type Rubisco form IB from *N. tabacum* (Stotz et al., 2011), *Larrea tridentata* (Henderson et al., 2011), and *A. thaliana* (Hasse et al., 2015) were solved. The sequences of these activases are longer than those of the Rca enzymes described above. In addition to the AAA+ core module, they feature a small domain at the N-terminus (N-domain) and a C-terminal extension, not resolved in the crystal structures (Figures 5A,B). The N-domain is required for targeting Rca to Rubisco (Esau et al., 1996; van de Loo and Salvucci, 1996; Stotz et al., 2011). It cooperates with a short helix (H9) in the α-helical subdomain of the AAA+ module, referred to as the specificity helix (Li et al., 2005; Stotz et al., 2011) (Figures 5B,D). In *N. tabacum* helix H9 interacts with residues arginine 89 and lysine 94 of RbcL (*N. tabacum* numbering) located in the equatorial region of the Rubisco complex and allows Rca to distinguish between solanaceous and non-solanaceous Rubisco (Portis et al., 2008; Wachter et al., 2013) (Figure 5D). The C-terminal extension is critical for the constitutive ATPase activity and mutation of tyrosine 361 results in loss of the ATPase and activase function (Stotz et al., 2011). Higher plants, including *A. thaliana*, rice, barley, maize and cotton, express two quasi-identical Rca isoforms, α and β, with the α-isoform possessing a slightly longer C-terminal extension (Portis et al., 2008). The isoforms are either expressed from separate genes or result from alternate splicing. The long C-terminal extension of the α-isoform contains two cysteine residues that can undergo F-type thioredoxin-dependent reversible oxidation (Zhang and Portis, 1999). Under oxidizing conditions, generally at night in the absence of photosynthesis, disulphide bond formation in the C-terminal extension inhibits ATP binding and thus Rubisco activation (Shen and Ogren, 1992; Zhang and Portis, 1999; Zhang et al., 2001, 2002; Portis, 2003; Wang and Portis, 2006; Portis et al., 2008; Carmo-Silva and Salvucci, 2013; Gontero and Salvucci, 2014).

Plant Rca enzymes have been reported to populate a range of dynamic oligomeric states *in vitro*, but are active as hexamers, as shown for the Rca enzymes of *N. tabacum* and *S. oleracea* (Blayney et al., 2011; Stotz et al., 2011; Keown and Pearce, 2014) (Figure 5C). Analysis of the NtRca by electron microscopy revealed the position of the N-domains at the top of the hexamer (Stotz et al., 2011). In the crystal structure of AtRca the N-domain was disordered (Hasse et al., 2015). Stable hexamers of NtRca were generated by mutation of arginine 294 to valine at the interface between adjacent subunits. Hexamers formed with ATP but not ADP and were functionally active (Stotz et al., 2011). In the case of cotton Rca, hexamer formation was also observed with ADP, but was less efficient than with ATP (Kuriata et al., 2014). Indeed, plant activases have been described to be sensitive to the ATP:ADP ratio (Portis et al., 2008; Carmo-Silva and Salvucci, 2013; Thieulin-Pardo et al., 2015). Such a regulation would ensure that Rca functions in a light- and redox-dependent (for the α-isoform) manner (Portis et al., 2008). Rca may also be functionally regulated by fluctuating Mg^2+^ concentrations in response to changes in available light, based on the finding that high Mg^2+^ caused an ~8-fold increase in catalytic activity of NtRca (Hazra et al., 2015).

The central pore of NtRca has a diameter of ~36 Å, wider than the Rca's described above (Mueller-Cajar et al., 2011; Stotz et al., 2011; Hasse et al., 2015; Sutter et al., 2015; Tsai et al., 2015) (Figures 3–5). NtRca and AtRca do not contain the canonical pore loop motif (aromatic-hydrophobic-glycine). Instead, three conserved loop segments face the central solvent channel and mutational analysis of NtRca implicates all of them in Rubisco remodeling (Stotz et al., 2011). This is similar to findings with the microtubule severing AAA+ protein spastin (Roll-Mecak and Vale, 2008). Based on the currently available structural and biochemical data, NtRca recognizes the inhibited Rubisco via the N-domain, with species specificity being imparted by helix H9. Notably, the RbcL of the green-type Rubisco form IB lacks the extended C-terminus that is required for the remodeling of red-type Rubisco. Thus, the exact mechanism of remodeling of plant Rubisco remains to be established.

## Convergent evolution of rubisco activase enzymes

It is believed that Rubisco-mediated CO_2_ fixation evolved ~3.5 billion years ago under non-oxygenic conditions (Nisbet et al., 2007). The evolution of cyanobacteria ~2.5 billion years ago triggered the shift to an oxygenic atmosphere (Whitney et al., 2011). During this process Rubisco also evolved into multiple enzymatic forms with a range of kinetic properties (Tcherkez et al., 2006; Badger and Bek, 2008; Sharwood et al., 2016; Young et al., 2016). Some Rubiscos apparently acquired mutations that led to tighter binding of RuBP and inhibitory sugar phosphates in the active site, necessitating the repair function by Rca. Notably, no sugar phosphate inhibition has been shown for cyanobacterial Rubiscos, although cyanobacteria contain genes encoding Rca-like proteins (Li et al., 1993), which are required for normal cell growth and Rubisco activity (Li et al., 1999). Interestingly, these proteins contain a C-terminal RbcS-like domain, which may mediate binding to Rubisco.

Recent studies have shown Rca's to exist also in prokaryotic and other eukaryotic organisms containing Rubiscos of form IA, IC, and ID (Figure 2B). The divergence in primary sequence of these proteins from different organisms strongly suggests that a process of convergent evolution underlies the use of the common AAA+ module in the Rubisco repair mechanism. Clearly, Rubiscos have co-evolved with their cognate activases, as exemplified by the C-terminal extension in red-type RbcL or the specific surface residues of solanaceous and non-solanaceous RbcL proteins that are recognized by their cognate activases (Wachter et al., 2013) (Figure 2B).

## Concluding remarks

Based on recent insights into the structural and functional diversity of Rubisco activases, these proteins represent an important paradigm to understanding how the AAA+ module can be adapted to the repair of a specific enzyme. Despite major progress, the exact mechanisms of remodeling are not yet understood. Which conformational changes does Rubisco undergo during reactivation? Are these effects limited to the active site pocket or are they more global? How does Rca distinguish between inhibited and active Rubisco? How is Rubisco remodeling reflected in the allostery of ATP binding and hydrolysis of the Rca subunits? Increasingly sophisticated biophysical techniques, such as hydrogen/deuterium exchange analysis and high resolution cryo-electron microscopy, should be brought to bear on these questions. Elucidating the mechanism of the plant Rca will be of special importance in the context of efforts to improve Rubisco carboxylation efficiency in crop plants (Whitney et al., 2011; Bracher et al., 2017). Engineering Rca itself may be a possible strategy, given its inherent thermal instability (Sage et al., 2008; Parry et al., 2013; Carmo-Silva et al., 2015). More likely, Rubisco and Rca may have to be co-engineered, mimicking the process that occurred during natural evolution.

## Author contributions

All authors listed, have made substantial, direct and intellectual contribution to the work, and approved it for publication.

### Conflict of interest statement

The authors declare that the research was conducted in the absence of any commercial or financial relationships that could be construed as a potential conflict of interest.

## References

[B1] Andersson I. (2008). Catalysis and regulation in Rubisco. J. Exp. Bot. 59, 1555–1568. 10.1093/jxb/ern091 18417482

[B2] Andersson I. Backlund A. (2008). Structure and function of Rubisco. Plant Physiol. Biochem. 46, 275–291. 10.1016/j.plaphy.2008.01.001 18294858

[B3] Andralojc P. J. Madgwick P. J. Tao Y. Keys A. Ward J. L. Beale M. H.. (2012). 2-Carboxy-D-arabinitol 1-phosphate (CA1P) phosphatase: evidence for a wider role in plant Rubisco regulation. Biochem. J. 442, 733–742. 10.1042/BJ20111443 22132794

[B4] Badger M. R. Bek E. J. (2008). Multiple Rubisco forms in proteobacteria: their functional significance in relation to CO_2_ acquisition by the CBB cycle. J. Exp. Bot. 59, 1525–1541. 10.1093/jxb/erm297 18245799

[B5] Blayney M. J. Whitney S. M. Beck J. L. (2011). NanoESI mass spectrometry of Rubisco and Rubisco activase structures and their interactions with nucleotides and sugar phosphates. J. Am. Soc. Mass Spectrom. 22, 1588–1601. 10.1007/s13361-011-0187-8 21953262

[B6] Bloom A. J. (2015). Photorespiration and nitrate assimilation: a major intersection between plant carbon and nitrogen. Photosyn. Res. 123, 117–128. 10.1007/s11120-014-0056-y 25366830

[B7] Bracher A. Whitney S. M. Hartl F. U. Hayer-Hartl M. (2017). Biogenesis and metabolic maintenance of Rubisco. Annu. Rev. Plant Biol. [Epub ahead of print]. 10.1146/annurev-arplant-043015-111633 28125284

[B8] Carmo-Silva A. E. Salvucci M. E. (2013). The regulatory properties of Rubisco activase differ among species and affect photosynthetic induction during light transitions. Plant Physiol. 161, 1645–1655. 10.1104/pp.112.213348 23417088 PMC3613445

[B9] Carmo-Silva E. Scales J. C. Madgwick P. J. Parry M. A. (2015). Optimizing Rubisco and its regulation for greater resource use efficiency. Plant Cell Environ. 38, 1817–1832. 10.1111/pce.12425 25123951

[B10] Cleland W. W. Andrews T. J. Gutteridge S. Hartman F. C. Lorimer G. H. (1998). Mechanism of Rubisco: the carbamate as general base. Chem. Rev. 98, 549–562. 10.1021/cr970010r 11848907

[B11] Duff A. P. Andrews T. J. Curmi P. M. (2000). The transition between the open and closed states of Rubisco is triggered by the inter-phosphate distance of the bound bisphosphate. J. Mol. Biol. 298, 903–916. 10.1006/jmbi.2000.3724 10801357

[B12] Ellis R. J. (1979). The most abundant protein in the world. Trends Biochem. Sci. 4, 241–244. 10.1016/0968-0004(79)90212-3

[B13] Esau B. D. Snyder G. W. Portis A. R. Jr. (1996). Differential effects of N- and C-terminal deletions on the two activities of Rubisco activase. Arch. Biochem. Biophys. 326, 100–105. 10.1006/abbi.1996.0052 8579356

[B14] Feller U. Anders I. Mae T. (2008). Rubiscolytics: fate of Rubisco after its enzymatic function in a cell is terminated. J. Exp. Bot. 59, 1615–1624. 10.1093/jxb/erm242 17975207

[B15] Field C. B. Behrenfeld M. J. Randerson J. T. Falkowski P. (1998). Primary production of the biosphere: integrating terrestrial and oceanic components. Science 281, 237–240. 10.1126/science.281.5374.237 9657713

[B16] Gibson J. L. Tabita F. R. (1997). Analysis of the *cbbXYZ* operon in *Rhodobacter sphaeroides*. J. Bacteriol. 179, 663–669. 10.1128/jb.179.3.663-669.1997 9006018 PMC178745

[B17] Gontero B. Salvucci M. E. (2014). Regulation of photosynthetic carbon metabolism in aquatic and terrestrial organisms by Rubisco activase, redox-modulation and CP12. Aquat. Bot. 118, 14–23. 10.1016/j.aquabot.2014.05.011

[B18] Hagemann M. Bauwe H. (2016). Photorespiration and the potential to improve photosynthesis. Curr. Opin. Chem. Biol. 35, 109–116. 10.1016/j.cbpa.2016.09.014 27693890

[B19] Hanson P. I. Whiteheart S. W. (2005). AAA+ proteins: have engine, will work. Nat. Rev. Mol. Cell Biol. 6, 519–529. 10.1038/nrm1684 16072036

[B20] Hasse D. Larsson A. M. Andersson I. (2015). Structure of *Arabidopsis thaliana* Rubisco activase. Acta Crystallogr. D Biol. Crystallogr. 71, 800–808. 10.1107/S1399004715001182 25849391

[B21] Hauser T. Popilka L. Hartl F. U. Hayer-Hartl M. (2015). Role of auxiliary proteins in Rubisco biogenesis and function. Nat. Plants 1:15065. 10.1038/nplants.2015.65 27250005

[B22] Hazra S. Henderson J. N. Liles K. Hilton M. T. Wachter R. M. (2015). Regulation of Ribulose-1,5-bisphosphate carboxylase/oxygenase (Rubisco) activase: product inhibition, cooperativity, and magnesium activation. J. Biol. Chem. 290, 24222–24236. 10.1074/jbc.M115.651745 26283786 PMC4591810

[B23] Henderson J. N. Kuriata A. M. Fromme R. Salvucci M. E. Wachter R. M. (2011). Atomic resolution X-ray structure of the substrate recognition domain of higher plant ribulose-bisphosphate carboxylase/oxygenase (Rubisco) activase. J. Biol. Chem. 286, 35683–35688. 10.1074/jbc.C111.289595 21880724 PMC3195603

[B24] Huson D. H. Scornavacca C. (2012). Dendroscope 3: an interactive tool for rooted phylogenetic trees and networks. Syst. Biol. 61, 1061–1067. 10.1093/sysbio/sys062 22780991

[B25] Jordan D. B. Chollet R. (1983). Inhibition of ribulose bisphosphate carboxylase by substrate ribulose 1,5-bisphosphate. J. Biol. Chem. 258, 13752–13758. 6417133

[B26] Keown J. R. Pearce F. G. (2014). Characterization of spinach ribulose-1,5-bisphosphate carboxylase/oxygenase activase isoforms reveals hexameric assemblies with increased thermal stability. Biochem. J. 464, 413–423. 10.1042/BJ20140676 25247706

[B27] Kerfeld C. A. Sawaya M. R. Pashkov I. Cannon G. Williams E. Tran K. (2004). The structure of *Halothiobacillus neapolitanus* Rubisco. 10.2210/pdb1svd/pdb

[B28] Kuriata A. M. Chakraborty M. Henderson J. N. Hazra S. Serban A. J. Pham T. V.. (2014). ATP and magnesium promote cotton short-form ribulose-1,5-bisphosphate carboxylase/oxygenase (Rubisco) activase hexamer formation at low micromolar concentrations. Biochemistry 53, 7232–7246. 10.1021/bi500968h 25357088

[B29] Li C. Salvucci M. E. Portis A. R. Jr. (2005). Two residues of Rubisco activase involved in recognition of the Rubisco substrate. J. Biol. Chem. 280, 24864–24869. 10.1074/jbc.M503547200 15866868

[B30] Li L. A. Gibson J. L. Tabita F. R. (1993). The Rubisco activase *(rca)* gene is located downstream from *rbcS* in *Anabaena* sp. strain CA and is detected in other Anabaena/Nostoc strains. Plant Mol. Biol. 21, 753–764. 10.1007/BF00027109 8467074

[B31] Li L. A. Zianni M. R. Tabita F. R. (1999). Inactivation of the monocistronic *rca* gene in *Anabaena variabilis* suggests a physiological ribulose bisphosphate carboxylase/oxygenase activase-like function in heterocystous cyanobacteria. Plant Mol. Biol. 40, 467–478. 10.1023/A:1006251808625 10437830

[B32] Loganathan N. Tsai Y. C. Mueller-Cajar O. (2016). Characterization of the heterooligomeric red-type Rubisco activase from red algae. Proc. Natl. Acad. Sci. U.S.A. 113, 14019–14024. 10.1073/pnas.1610758113 27872295 PMC5150372

[B33] Miziorko H. M. Lorimer G. H. (1983). Ribulose-1,5-bisphosphate carboxylase-oxygenase. Annu. Rev. Biochem. 52, 507–535. 10.1146/annurev.bi.52.070183.002451 6351728

[B34] Mueller-Cajar O. Stotz M. Wendler P. Hartl F. U. Bracher A. Hayer-Hartl M. (2011). Structure and function of the AAA+ protein CbbX, a red-type Rubisco activase. Nature 479, 194–199. 10.1038/nature10568 22048315

[B35] Neuwald A. F. Aravind L. Spouge J. L. Koonin E. V. (1999). AAA+: a class of chaperone-like ATPases associated with the assembly, operation, and disassembly of protein complexes. Genome Res. 9, 27–43. 9927482

[B36] Nisbet E. G. Grassineau N. V. Howe C. J. Abell P. I. Regelous M. Nisbet R. E. R. (2007). The age of Rubisco: the evolution of oxygenic photosynthesis. Geobiology 5, 311–335. 10.1111/j.1472-4669.2007.00127.x

[B37] Notredame C. Higgins D. G. Heringa J. (2000). T-Coffee: a novel method for fast and accurate multiple sequence alignment. J. Mol. Biol. 302, 205–217. 10.1006/jmbi.2000.4042 10964570

[B38] Olivares A. O. Baker T. A. Sauer R. T. (2016). Mechanistic insights into bacterial AAA+ proteases and protein-remodelling machines. Nat. Rev. Microbiol. 14, 33–44. 10.1038/nrmicro.2015.4 26639779 PMC5458636

[B39] Parry M. A. Andralojc P. J. Scales J. C. Salvucci M. E. Carmo-Silva A. E. Alonso H.. (2013). Rubisco activity and regulation as targets for crop improvement. J. Exp. Bot. 64, 717–730. 10.1093/jxb/ers336 23162118

[B40] Parry M. A. Keys A. J. Madgwick P. J. Carmo-Silva A. E. Andralojc P. J. (2008). Rubisco regulation: a role for inhibitors. J. Exp. Bot. 59, 1569–1580. 10.1093/jxb/ern084 18436543

[B41] Pearce F. G. (2006). Catalytic by-product formation and ligand binding by ribulose bisphosphate carboxylases from different phylogenies. Biochem. J. 399, 525–534. 10.1042/BJ20060430 16822231 PMC1615894

[B42] Portis A. R. Jr. (2003). Rubisco activase–Rubisco's catalytic chaperone. Photosynth. Res. 75, 11–27. 10.1023/A:1022458108678 16245090

[B43] Portis A. R. Jr. Li C. Wang D. Salvucci M. E. (2008). Regulation of Rubisco activase and its interaction with Rubisco. J. Exp. Bot. 59, 1597–1604. 10.1093/jxb/erm240 18048372

[B44] Portis A. R. Jr. Salvucci M. E. (2002). The discovery of Rubisco activase–yet another story of serendipity. Photosynth. Res. 73, 257–264. 10.1023/A:1020423802875 16245129

[B45] Roll-Mecak A. Vale R. D. (2008). Structural basis of microtubule severing by the hereditary spastic paraplegia protein spastin. Nature 451, 363–367. 10.1038/nature06482 18202664 PMC2882799

[B46] Sage R. F. Way D. A. Kubien D. S. (2008). Rubisco, Rubisco activase, and global climate change. J. Exp. Bot. 59, 1581–1595. 10.1093/jxb/ern053 18436544

[B47] Sharwood R. E. Ghannoum O. Whitney S. M. (2016). Prospects for improving CO_2_ fixation in C_3_-crops through understanding C_4_-Rubisco biogenesis and catalytic diversity. Curr. Opin. Plant Biol. 31, 135–142. 10.1016/j.pbi.2016.04.002 27131319

[B48] Shen J. B. Ogren W. L. (1992). Alteration of spinach ribulose-1,5-bisphosphate carboxylase/oxygenase activase activities by site-directed mutagenesis. Plant Physiol. 99, 1201–1207. 10.1104/pp.99.3.1201 16668989 PMC1080603

[B49] Snider J. Thibault G. Houry W. A. (2008). The AAA+ superfamily of functionally diverse proteins. Genome Biol. 9:216. 10.1186/gb-2008-9-4-216 18466635 PMC2643927

[B50] Stec B. (2012). Structural mechanism of Rubisco activation by carbamylation of the active site lysine. Proc. Natl. Acad. Sci. U.S.A. 109, 18785–18790. 10.1073/pnas.1210754109 23112176 PMC3503183

[B51] Stotz M. Mueller-Cajar O. Ciniawsky S. Wendler P. Hartl F. U. Bracher A.. (2011). Structure of green-type Rubisco activase from tobacco. Nat. Struct. Mol. Biol. 18, 1366–1370. 10.1038/nsmb.2171 22056769

[B52] Sutter M. Roberts E. W. Gonzalez R. C. Bates C. Dawoud S. Landry K.. (2015). Structural characterization of a newly identified component of alpha-carboxysomes: the AAA+ domain protein CsoCbbQ. Sci. Rep. 5:16243. 10.1038/srep16243 26538283 PMC4633670

[B53] Taylor T. C. Andersson I. (1997). The structure of the complex between Rubisco and its natural substrate ribulose 1,5-bisphosphate. J. Mol. Biol. 265, 432–444. 10.1006/jmbi.1996.0738 9034362

[B54] Tcherkez G. G. Farquhar G. D. Andrews T. J. (2006). Despite slow catalysis and confused substrate specificity, all ribulose bisphosphate carboxylases may be nearly perfectly optimized. Proc. Natl. Acad. Sci. U.S.A. 103, 7246–7251. 10.1073/pnas.0600605103 16641091 PMC1464328

[B55] Thieulin-Pardo G. Avilan L. Kojadinovic M. Gontero B. (2015). Fairy “tails”: flexibility and function of intrinsically disordered extensions in the photosynthetic world. Front. Mol. Biosci. 2:23. 10.3389/fmolb.2015.00023 26042223 PMC4436894

[B56] Tsai Y. C. Lapina M. C. Bhushan S. Mueller-Cajar O. (2015). Identification and characterization of multiple Rubisco activases in chemoautotrophic bacteria. Nat. Commun. 6:8883. 10.1038/ncomms9883 26567524 PMC4660213

[B57] van de Loo F. J. Salvucci M. E. (1996). Activation of ribulose-1,5-biphosphate carboxylase/oxygenase (Rubisco) involves Rubisco activase Trp16. Biochemistry 35, 8143–8148. 10.1021/bi9604901 8679566

[B58] Von Caemmerer S. Edmondson D. L. (1986). Relationship between steady-state gas exchange *in vivo* ribulose bisphosphate carboxylase activity and some carbon reduction cycle intermediates in Raphanus sativus. Aust. J. Plant Physiol. 13, 669–688. 10.1071/PP9860669

[B59] Wachter R. M. Salvucci M. E. Carmo-Silva A. E. Barta C. Genkov T. Spreitzer R. J. (2013). Activation of interspecies-hybrid Rubisco enzymes to assess different models for the Rubisco-Rubisco activase interaction. Photosynth. Res. 117, 557–566. 10.1007/s11120-013-9827-0 23613007

[B60] Walker B. J. VanLoocke A. Bernacchi C. J. Ort D. R. (2016). The costs of photorespiration to food production now and in the future. Annu. Rev. Plant Biol. 67, 107–129. 10.1146/annurev-arplant-043015-111709 26865340

[B61] Wang D. Portis A. R. Jr. (2006). Increased sensitivity of oxidized large isoform of ribulose-1,5-bisphosphate carboxylase/oxygenase (Rubisco) activase to ADP inhibition is due to an interaction between its carboxyl extension and nucleotide-binding pocket. J. Biol. Chem. 281, 25241–25249. 10.1074/jbc.M604756200 16822862

[B62] Wendler P. Ciniawsky S. Kock M. Kube S. (2012). Structure and function of the AAA+ nucleotide binding pocket. Biochim. Biophys. Acta 1823, 2–14. 10.1016/j.bbamcr.2011.06.014 21839118

[B63] Whitney S. M. Houtz R. L. Alonso H. (2011). Advancing our understanding and capacity to engineer nature's CO_2_-sequestering enzyme, Rubisco. Plant Physiol. 155, 27–35. 10.1104/pp.110.164814 20974895 PMC3075749

[B64] Whittaker C. A. Hynes R. O. (2002). Distribution and evolution of von Willebrand/integrin A domains: widely dispersed domains with roles in cell adhesion and elsewhere. Mol. Biol. Cell 13, 3369–3387. 10.1091/mbc.E02-05-0259 12388743 PMC129952

[B65] Wong K. S. Houry W. A. (2012). Novel structural and functional insights into the MoxR family of AAA+ ATPases. J. Struct. Biol. 179, 211–221. 10.1016/j.jsb.2012.03.010 22491058

[B66] Young J. N. Heureux A. M. Sharwood R. E. Rickaby R. E. Morel F. M. Whitney S. M. (2016). Large variation in the Rubisco kinetics of diatoms reveals diversity among their carbon-concentrating mechanisms. J. Exp. Bot. 67, 3445–3456. 10.1093/jxb/erw163 27129950 PMC4892730

[B67] Zhang N. Kallis R. P. Ewy R. G. Portis A. R. Jr. (2002). Light modulation of Rubisco in *Arabidopsis* requires a capacity for redox regulation of the larger Rubisco activase isoform. Proc. Natl. Acad. Sci. U.S.A. 99, 3330–3334. 10.1073/pnas.042529999 11854454 PMC122518

[B68] Zhang N. Portis A. R. Jr. (1999). Mechanism of light regulation of Rubisco: a specific role for the larger Rubisco activase isoform involving reductive activation by thioredoxin-f. Proc. Natl. Acad. Sci. U.S.A. 96, 9438–9443. 10.1073/pnas.96.16.9438 10430961 PMC17801

[B69] Zhang N. Schürmann P. Portis A. R. Jr. (2001). Characterization of the regulatory function of the 46-kDa isoform of Rubisco activase from *Arabidopsis*. Photosynth. Res. 68, 29–37. 10.1023/A:1011845506196 16228326

[B70] Zhu G. Jensen R. G. (1991). Fallover of ribulose 1,5-bisphosphate carboxylase/oxygenase activity: decarbamylation of catalytic sites depends on pH. Plant Physiol. 97, 1354–1358. 10.1104/pp.97.4.1354 16668556 PMC1081171

